# Updates on molecular mechanisms in the development of branched trichome in Arabidopsis and nonbranched in cotton

**DOI:** 10.1111/pbi.13167

**Published:** 2019-06-11

**Authors:** Zhi Wang, Zuoren Yang, Fuguang Li

**Affiliations:** ^1^ Zhengzhou Research Base State Key Laboratory of Cotton Biology Zhengzhou University Zhengzhou China; ^2^ State Key Laboratory of Cotton Biology Institute of Cotton Research Chinese Academy of Agricultural Sciences Anyang China

**Keywords:** plant trichome, Arabidopsis, cotton, molecular mechanism

## Abstract

Trichomes are specialized epidermal cells and a vital plant organ that protect plants from various harms and provide valuable resources for plant development and use. Some key genes related to trichomes have been identified in the model plant *Arabidopsis thaliana* through glabrous mutants and gene cloning, and the hub MYB‐bHLH‐WD40, consisting of several factors including GLABRA1 (GL1), GL3, TRANSPARENT TESTA GLABRA1 (TTG1), and ENHANCER OF GLABRA3 (EGL3), has been established. Subsequently, some upstream transcription factors, phytohormones and epigenetic modification factors have also been studied in depth. In cotton, a very important fibre and oil crop globally, in addition to the key MYB‐like factors, more important regulators and potential molecular mechanisms (e.g. epigenetic modifiers, distinct metabolic pathways) are being exploited during different fibre developmental stages. This occurs due to increased cotton research, resulting in the discovery of more complex regulation mechanisms from the allotetraploid genome of cotton. In addition, some conservative as well as specific mediators are involved in trichome development in other species. This study summarizes molecular mechanisms in trichome development and provides a detailed comparison of the similarities and differences between Arabidopsis and cotton, analyses the possible reasons for the discrepancy in identification of regulators, and raises future questions and foci for understanding trichome development in more detail.

## Introduction

Plant trichomes are epidermal outgrowths that play multiple roles in plant development. Morphologically, trichomes are one of the key factors serving as a buffer zone between the plant surface and the environment, protecting plants from adverse environmental conditions and other external hazards like toxic chemicals and herbivorous insects (Mauricio and Rausher, 1997). For example, the trichomes of *Pteris vittata* can absorb the heavy metal arsenic and protect the plant from arsenic contamination (Li *et al*., 2005a). Genetic engineering of the cysteine biosynthesis pathway, involved by *AtCYS‐3A* in leaf trichomes, displays preferable heavy metal cadmium absorbing and phytoremediation effect in Arabidopsis (Dominguez‐Solis *et al*., 2004). The Venus flytrap (*Dionaea muscipula*) snares crickets and obtains nutrients from them depending on the sensing and signal transduction of the leaf trichomes (Forterre *et al*., 2005; Stokstad, 2016). In addition, trichomes generate and store some useful and valuable chemical molecules that are important resources not only for the plant development and defence but also for human living or disease treatment. For instance, trichomes in the seed coat of cotton, also referred to as fibre, are an important source and raw material for the natural fibre and textile industry due to their high cellulose content (Rinehart *et al*., 1996). In other species such as *Humulus lupulus*,* Mentha* spp. and *Artemisia annua*, the trichome is important for the synthesis of polysaccharides, proteins, polyphenols and terpenoids, which can be used for the extraction of medicine, herbicide, food additives or resin (Lange and Turner, 2013; Singh *et al*., 2016). Trichomes of *A. annua* can produce artemisinin, the well‐known antimalarial drug that is a kind of sesquiterpenoid (Singh *et al*., 2016). Trichomes are thus also regarded as mini chemical ‘factories’ for high‐value natural products similar to fibre (Akhtar *et al*., 2017; Bryant *et al*., 2016; Champagne and Boutry, 2017; Wang *et al*., 2016b).

Morphologically, as giant single epidermal cells, trichomes are easy to analyse at the genetic, genomic and cell biology levels and have thus turned into a model system for research in cell development. Trichome development is typically studied using Arabidopsis rosette leaves or the cotton fibre. In Arabidopsis, further trichome division occurs due to cell divisions of pavement cells in the middle of the epidermis. The differentiating trichomes undergo four endoreduplication cycles, typically resulting in three or four branches (Akhtar *et al*., 2017). Cell‐cycle regulation and cell morphogenesis are heavily studied in trichomes using certain mutants (Haigler *et al*., 2012; Jakoby *et al*., 2008; Marks *et al*., 2008).

The evidence from trichome mutants has shown that trichome development is impeded at various developmental stages, indicating that trichome development is sophisticated and intricate in plants (Hulskamp, 2004; Smith and Oppenheimer, 2005; Szymanski *et al*., 2000), which is different from the trichome development in Arabidopsis. As a unicellular organ, the trichomes on cotton seed coats have four continuous but overlapping developmental stages: initiation, elongation, secondary cell wall (SCW) deposition and maturation (Haigler *et al*., 2012). Due to the importance of fibre in human life and the textile industry, multiple genes and potential functions related to fibre yield and quality have been identified and elucidated in cotton.

In this review, the underlying molecular mechanisms and key factors for trichome development in Arabidopsis and cotton are summarized and some potential future research areas are suggested to provide an understanding of how trichome development is regulated.

## The regulation mechanisms of leaf trichome development in Arabidopsis

In Arabidopsis, the trichomes in rosette leaves have been widely studied. Studies have shown that trichomes are derived from rapidly dividing proepidermal cells in new leaf bases (Larkin *et al*., 1993). After quartic endoreduplications, the trichome cells mature and form 2–4 branches depending on the geographic location of the plant species (Hulskamp *et al*., 1994). Through mutant and genetic analysis, many more genes involved in trichome development have been identified and studied (Larkin *et al*., 1994; Schellmann *et al*. 2002; Wan *et al*., 2016; Wu *et al*., 2018; Zhang *et al*., 2003).

## The key transcription factors underlying trichome development in Arabidopsis

GLABRA1 (GL1), the earliest identified regulator in plants trichome development, encodes a MYB‐like protein, knockout of which results in glabrous leaves (Larkin *et al*., 1994). However, over‐expression of *GL1* also decreases trichome identity, possibly because the leaf epidermal inhibition programme (LEIP) but not the gene cosuppression system is activated by over‐expressing *GL1*. This indicates the vital roles of homeostasis of GL1 and the delicate regulation web involving GL1 in trichome differentiation and development (Larkin *et al*., 1994; Szymanski *et al*., 1999). However, researchers still do not have detailed information about the LEIP. *GL2* is a homeobox family gene, encoding an HD‐ZIP IV domain transcriptional factor, which is necessary for trichome development. The knockout mutant *gl2* displayed no trichomes in the first pair of true leaves and decreased trichomes in other leaves as well as no branches in most trichomes (Johnson *et al*., 2002; Szymanski *et al*., 1998).

**Figure 1:**
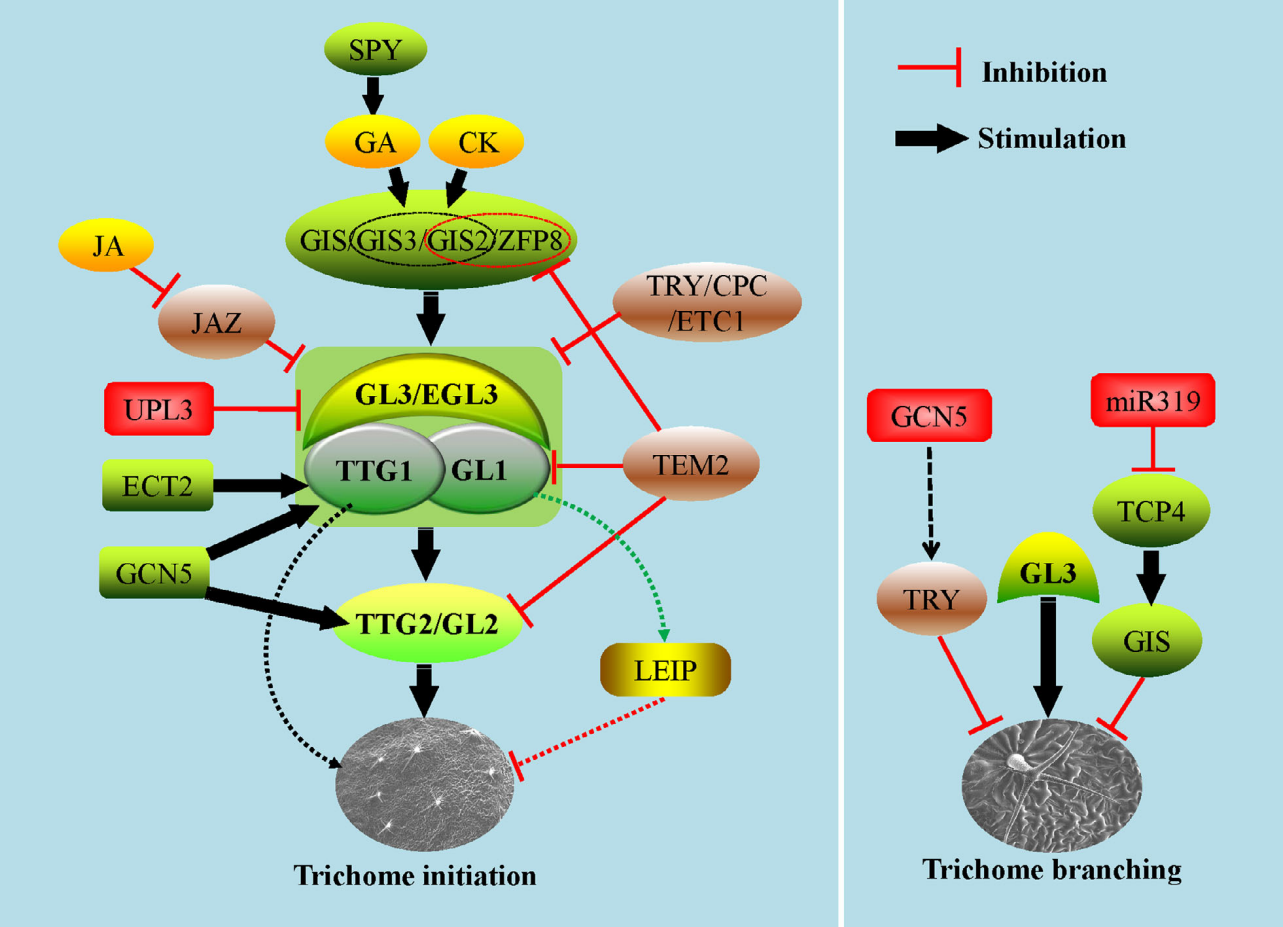
The positive and negative regulation through hub in Arabidopsis trichome development. Left panel, the regulation in trichome initiation. The hub comprises GL1, TTG1, GL3 and EGL3, in which EGL3 function redundantly with GL3. Some redundant regulators including GIS, GIS2, GIS3 and ZFP8 function upstream of the hub as important mediators, linking GA and CK to regulate trichome development. GL2 functions downstream of the hub in trichome development and partially redundant with TTG2. Green ovals indicate the positive transcription factors. Yellow ovals indicate the stimulating phytohormones. The brown oval indicates the negative transcription factor upstream of the hub. The green and red rounded rectangles indicate positive and negative epigenetic factors, among which ECT2 stabilizes TTG1 transcripts and positively regulates trichomes. TEM2 represses both GL1 and GL2 transcriptionally to inhibit trichome development directly. Further, TEM2 also represses GIS2 and ZFP8 directly by binding their promoters. GL3 and EGL3 are degraded through protein ubiquitination pathway involved by UPL3 directly. Moreover, over‐expression of GL1 repressed trichome development through triggering an unknown leaf epidermal inhibition programme (LEIP) (green and red dashes). Therefore, the homeostasis of GL1 is vital for trichome development in Arabidopsis. The yellow oval – JA degrades transcription factor JAZ and in turn activates the GL1/TTG1/GL3 hub in trichome development. The green rounded rectangle – GCN5 promotes GL1, GL2 and GL3 to involve trichome initiation positively. Because mutant *gl2* showed less defects in trichome development than *gl1* and *ttg1* mutants, other unknown pathways downstream of GL1/TTG1 may exist in trichome development (black dashes). Right panel, the regulation in trichome branching. The red rounded rectangle – GCN5 represses TRY function indirectly in trichome branching. Another red rounded rectangle indicates the inhibiting epigenetic modifier‐miR319, which down‐regulated TCP4 by microRNA‐mediating RNA interfering pathway and GIS involved in trichome branching. GIS is a multifunctional regulator, playing positive and negative roles in trichome initiation and branching, respectively. In addition, GL3 also plays positive role in trichome branching.

*GL3* encodes a typical bHLH transcription factor, which mainly regulates the branches, endoreduplication and epidermal cell size (Payne *et al*., 2000; Shen *et al*., 2006; Szymanski *et al*., 2000). ENHANCER OF GLABRA3 (EGL3), another bHLH type transcription factor, had a redundant role with GL3, and the double mutant of GL3 and EGL3 resulted in absolute trichome defect (Zhang *et al*. 2003). TRANSPARENT TESTA GLABRA1 (TTG1) encodes a small protein with 4–5 repeat WD‐40 motifs, which can interact with GL3 to positively influence trichome differentiation (Payne *et al*., 2000). Hypomorphic alleles of GL1 and TTG1 produced aborted trichomes, and interactions between *gl1* and *ttg1* displayed a fixed effect in clustering trichomes as in single mutants, which suggests that GL1 and TTG1 function as a complex and as dual regulators in trichome development (Larkin *et al*., 1999). TTG2, encoding a WRKY transcription factor, acts downstream of TTG1 and GL1 and has redundant effects with GL2 in regulating trichome outgrowth (Johnson *et al*., 2002) (Figure 1). So, TTG2 and GL2 could complement each other to regulate downstream targets and trichome development when plant encounters sudden environment change or external invasion.

SENSITIVE TO ABA AND DROUGHT2 (SAD2) encodes an important beta‐domain protein, which regulates trichome development in the same way as GL1, GL2 and GL3 genetically. However, SAD2 does not influence the interaction between GL3 and TTG1 (Gao *et al*., 2008; Verslues *et al*., 2006) (Figure 1); the detailed interaction and molecular mechanisms between SAD2 and the core transcription factors (GL1, GL3, TTG1) are unknown. TRIPTYCHON (TRY) and CAPRICE (CPC) are two negative and redundant regulators in trichome development, both encoding MYB‐type transcription factors. TRY and CPC can bind the N terminus of GL3 and EGL3 competitively to impair the function of the complex associated with GL3 and EGL3, then disturb trichome differentiation and development (Kirik *et al*., 2004; Larkin *et al*., 2003; Schellmann *et al*., 2002; Zhang *et al*. 2003). ENHANCER OF TRY AND CPC1 (ETC1) is similar to TRY and CPC in sequence and function and plays a partially redundant role with TRY and CPC in repressing trichome initiation (Kirik *et al*., 2004; Figure 1).

Genetic data of the interaction between *gl2*,* gl3* and *try* provide more substantial evidence that GL2 and GL3 have redundant roles in mediating genes required in trichome morphogenesis; TRY and GL2 may function partly independently in trichome regulation (Hulskamp *et al*., 1994). The biochemical data further support direct interaction and binding among GL1, GL3, TTG1, TRY and CPC (Kirik *et al*., 2004; Payne *et al*., 2000; Zhang *et al*., 2003). In summary, an active‐repressive system comprising some transcription factors serves as a hub in trichome differentiation and development, in which MYB‐bHLH‐WD40 and TRY/CPC play positive and negative roles, respectively (Figure 1). These findings give us a core model of regulation of trichome development in Arabidopsis, but the full, detailed regulation network remains elusive. Interestingly, there are so many types of transcription factors in plant, why only the above transcription factors regulate the trichome development from the updated research, and whether other types of transcription factors are also involved in the trichome development is not clear. Some potential and specific protein domains for trichome development may be explored and studied from the known MYB‐bHLH‐WD40 transcription factors.

## Associated hormonal mechanisms underlying trichome development in Arabidopsis

The development of trichomes is tightly regulated by integrated environmental and endogenous signals. Phytohormones (e.g. auxin, gibberellic acids (GAs), cytokines (CK), jasmonic acid (JA) and brassinosteroids (BRs)) function as important signals in trichome development by mediating downstream genes; some related factors have also been identified in the regulation network. GAs are required for trichome proliferation in rosette leaves, stem and inflorescence (An *et al*., 2012; Perazza *et al*., 1998). Mutation of GA synthesis factor GAI and the GA signalling repressor SPINDLY (SPY) significantly affected trichome development, which showed the positive correlation between GA levels and trichome development. Genetic analysis indicated that GAs facilitate trichome development dependent on *GL1* and possibly TTG (Jacobsen *et al*., 1996; Perazza *et al*., 1998).

*GLABROUS INFLORESCENCE STEMS* (*GIS*), encoding a C2H2 transcription factor in trichome initiation pathway, positively regulates the trichome hub formed by GL1, GL3, EGL3 and TTG1 in the epidermis by the GA pathway (Payne *et al*., 2000; Zhao *et al*., 2008). But, on the other hand, GIS also has a negative function in trichome branching (An *et al*., 2012), which indicates that GIS is a central and multifunctional regulator in trichome development. Furthermore, GIS2 and ZINC FINGER PROTEIN8 (ZFP8), two other C2H2 transcription factors, are necessary in trichome production involving the CK pathway (Gan *et al*., 2007). GIS2, GIS3 and ZFP8 regulate trichome initiation by the GA pathway like GIS. However, GIS is not involved in the CK pathway; therefore, equivalent proteins such as GIS, GIS2, GIS3 and ZFP8 perform partially redundant functions in trichome initiation and result in crosstalk of CK and GA (Figure 1; Gan *et al*., 2007; Sun *et al*., 2015a).

TEMPRANILLO1 (TEM1) and TEM2 encode members of the RELATED TO ABI3 AND VP1 transcription factor family. RAV is plant‐specific, and TEM1 and TEM2 show negative roles in trichome development dependent on phytohormone GA and CK pathways. More work showed TEMs regulate not only GA content but also GA transport and distribution in the leaf mesophyll, in turn mediate the trichome development programme in the epidermis (Matias‐Hernandez *et al*., 2016), indicating the roles of cells underneath the epidermis in trichome initiation. Chromatin immunoprecipitation (ChIP) also showed direct binding of TEMs on the promoters of GL1, GL2, GIS2 and ZFP8 (Matias‐Hernandez *et al*., 2016), which indicated possible transcription repression and upstream regulation of TEMs on some transcription factors encoding genes in trichome initiation. JASMONATE‐ZIM‐DOMAIN1 (JAZ1) protein, an important repressor in the JA signalling pathway, can be degraded by JA, releasing MYB‐bHLH‐WD40 activity and promoting trichome development (Qi *et al*., 2011). These results make the underlying mechanisms of key transcription factors more concrete, suggesting a well‐arranged and orchestrated transcription regulation network associated with phytohormones in trichome development (Figure 1).

## Epigenetic modifications underlying trichome development in Arabidopsis

Epigenetic modifications are a vital mode of regulation and are mainly involved in numerous protein post‐translational modifications, such as ubiquitination, acetylation, methylation, sumoylation, glycosylation and DNA methylation. In addition, noncoding RNAs also play important roles in plant development as epigenetic regulators (Boyko and Kovalchuk, 2008; Davila‐Velderrain *et al*., 2018; Grant‐Downton and Dickinson, 2005; Steimer *et al*., 2004; Yamamuro *et al*., 2016). A recent study showed that a multifunctional histone acetyltransferase, AtGCN5, positively influences trichome branching with possible regulation of TRY (Kotak *et al*., 2018). On the other hand, through regulating histone acetylation of the promoters of GL1, GL2, GL3 and CPC, GCN5 is also involved trichome initiation regulation (Wang *et al*., 2019b). Cell‐free degradation, *in planta* assays, and mutant analysis showed that ubiquitin protein ligase3 (UPL3) promoted the degradation of GL3 and EGL3, which in turn inhibited trichome branching (Patra *et al*., 2013) (Figure 1).

As a histone chaperone, chromatin assembly factor‐1 (CAF‐1) is involved in trichome development *via* an endoreduplication‐independent pathway (Exner *et al*., 2008). mRNA modifications are associated with cell differentiation and development in eukaryotes, of which N6‐methyladenosine (m6A) is the most prevalent epitranscriptomic mark (Meyer *et al*., 2012; Wang *et al*., 2014b). Similar to DNA methylation, m6A can be written, erased and read by different factors known to regulate gene expression and function (Bokar *et al*., 1997; Wang *et al*., 2015b; Xiao *et al*., 2016; Zheng *et al*., 2013). EVOLUTIONARILY CONSERVED C‐TERMINAL REGION2 (ECT2) was identified as an m6A reader protein and is involved in trichome development through binding and stabilizing some key transcripts related to trichomes including TTG1 (Wei *et al*., 2018; Figure 1), which provides new clue for the post‐transcriptional modification in the key factors in trichome development.

Noncoding RNAs are classified into several groups according to the lengths of mature transcripts. microRNAs (miRNAs) and small interfering RNAs (siRNAs) are 20–30 nucleotides (nt) in length; medium ncRNAs are 50–200 nt in length; and long noncoding RNAs (lncRNAs) are longer than 200 nt in mature transcripts (Liu *et al*., 2013). miRNA‐mediated gene silencing is one of the most important epigenetic modifications in plant development. Recently, miR319‐regulated PROLIFERATING CELL FACTOR4 (TCP4) protein was shown to suppress trichome branching by directly activating GIS transcription in Arabidopsis leaves (Vadde *et al*., 2018; Figure 1). These results indicated that epigenetic mechanisms including multilevel modifications in DNA, RNA and protein played important roles in trichome initiation and branching regulation.

Collectively, in trichome development of Arabidopsis, the hub consisting of GL1, GL3, EGL3 and TTGL1 is crucial; however, GL1 and TTG1 function as dual regulators involved in trichome morphogenesis and development. Surrounding them, many transcriptional regulators and signal molecules are involved in the trichome development through different pathways. Of these, GIS is a specific regulator, which plays positive and negative roles in trichome initiation and branching, respectively. The reason for the dual functions of GIS may be the conservation of energy and homeostasis between trichome differentiation and branching. However, researchers still do not fully understand the underlying mechanisms. As one of the pivotal and universal regulation mechanisms of gene and protein expression, epigenetic modifications have shown some roles in trichome development. However, many more studies are needed to uncover the specific and delicate interaction among epigenetic factors, phytohormones and the key trichome regulators.

## The regulation mechanisms of trichome development on the seed coat of cotton

Trichomes on the cotton seed coat, generally referred to as fibres, are the main harvest product of cotton and are a significant valuable resource for textile industries (Qin and Zhu, 2011). Much work has been conducted to elucidate the underlying mechanisms of the four classic stages of initiation, elongation, SCW and maturation in fibre development. The regulation mechanisms of fibre initiation and elongation are relatively better understood, since some genes that have been characterized are implicated in initiation and elongation (Du *et al*., 2018; Huang *et al*., 2017; Li *et al*., 2014, 2015a; Ma *et al*., 2018; Sun *et al*., 2017; Wen *et al*., 2018).

## The identification of conserved transcription factors in cotton fibre development

Regulation mechanisms involved in trichome development in cotton are similar to those in the model plant Arabidopsis. Some key homologous genes encoding MYB‐type transcription factors such as GL1 and CPC have been cloned and identified in fibre initiation (Liu *et al*., 2015a; Wang *et al*., 2004). By expressed sequence tag (EST) screening, 55 gene fragments expressed in early ovules were identified. RNAi transgenic plants showed that GhMYB109 was positively involved in trichome initiation and differentiation (Suo *et al*., 2003). Another MYB transcription factor, GhMYB25, was also identified to have an important role in fibre initiation and elongation by the glabrous mutant and transgenic assay (Machado *et al*., 2009). Using the naked mutant *N1*, lintless fuzzless mutant xuzhou142*fl*, and mapping cloning, two MYB25‐like transcriptional factors, GhMML3_A12 and GhMML4_D12, were identified as key regulators in fuzz fibre and lint fibre development, respectively (Wan *et al*., 2016; Wu *et al*., 2018). GhJAZ2 protein, a similar negative regulator in the JA pathway as that in Arabidopsis, interacted with GhMYB25‐like and GhGL1 to repress their functions and fibre development (Hu *et al*., 2016; Figure 3).

Recently, GhMYB212 was identified as an important regulator in the transport of sucrose from ovules to fibres during fibre elongation. GhMYB212 can directly control the expression of a sucrose transporter gene GhSWEET12 and in turn mediate the sucrose and glucose transport and fibre development (Sun *et al*., 2018). Moreover, Huang *et al*. (2017) identified 419 R2R3‐MYB in a systematic analysis of the cotton genome and demonstrated the roles of GhMYB46_D13 and GhMYB46_D9 in the fibre SCW deposition stage. The GL2 homologs in cotton, including GbML1 (MERISTEM LAYER 1), GhHD1 and homeoboxes (HOXs), have been cloned and identified in fibre development. Over‐expression of GbML1 increased the trichome density in leaf and stem in Arabidopsis (Zhang *et al*., 2010). Both RNAi and over‐expression of *GhHD1* showed that *GhHD1* plays positive roles in trichome initiation through mediating accumulation of ethylene and reactive oxygen species (ROS) (Walford *et al*., 2012; Zhang *et al*., 2010). Three cotton *HOX* genes have been cloned by Chen *et al*. (Guan *et al*., 2008; Wang *et al*., 2004). Detailed research showed that GaHOX1 can rescue the trichome initiation of *gl2‐2*; over‐expression and knockdown of GhHOX3 can significantly increase or decrease the fibre length, respectively (Guan *et al*., 2008; Shan *et al*., 2014).

Deeper study showed that the GhHOX3‐GhHD1 interaction increased GhHOX3 transcription activity and its role in fibre elongation. Furthermore, a cotton DELLA protein, GhSLR1, interfered with GhHOX3‐GhHD1 complex stability and repressed downstream target gene transcription and subsequent fibre elongation (Shan *et al*., 2014). In Arabidopsis, CPC interacts with and inhibits the function of the complex consisting of GL1, GL3, EGL3 and TTG1 involved in trichome initiation (Kirik *et al*., 2004). GhCPC was also identified to interact with GhMYC1 (GL3) to play a negative role in fibre initiation, which indicates a partially similar regulation model in cotton as in Arabidopsis (Liu *et al*., 2015a). Further, GhMYC1 can bind the E‐BOX in the promoter of GhHOX1, indicating that GhHOX1 may be downstream of GhMYC1 (GL3) in fibre regulation (Wan *et al*., 2016) (Figure 3). More recently, a NAC transcription factor, GhFSN1, was identified to play a positive role in the SCW deposition stage by binding and activating downstream SCW‐related genes (Zhang *et al*., 2018a). As noted above, many more transcription factors including MYB, bHLH, and homeobox families have been identified in trichomes on the seed coat of cotton than in Arabidopsis, indicating a likely complex regulation network in cotton fibre development.

## The underlying mechanisms of phytohormones in cotton fibre development

Phytohormones such as auxin, GA, ethylene, CK, BR and abscisic acid play important roles in fibre development (Kim *et al*., 2015; Perazza *et al*., 1998; Seagull and Giavalis, 2004; Sun *et al*., 2005, 2015b; Zhang *et al*., 2009a). However, the intricate mechanisms of these phytohormones are not yet clear. 1‐Aminocyclopropane‐1‐carboxylic acid oxidase (ACO) is responsible for ethylene synthesis as the last rate‐limiting enzyme. Three ACO encoding genes were expressed in the fibre elongation stage and had a positive correlation with fibre elongation, supporting the potential roles of ethylene in fibre development (Shi *et al*., 2006). In cotton, *PROTODERMAL FACTOR1* (*GbPDF1*), encoding a homeobox‐leucine zipper protein, was primarily expressed during fibre initiation and early elongation. Knockdown of *GbPDF1* resulted in a fibre initiation delay, fibre shortening and lint percentage decrease, indicating its essential role in trichome development. More work explored PDF1 regulation of H_2_O_2_ homeostasis involved in fibre development (Deng *et al*., 2012). Exogenous ethylene can promote H_2_O_2_ accumulation to positively mediate fibre development, indicating the synergistic interaction between ethylene and ROS pathways in fibre (Li *et al*., 2007). The underlying regulators and mechanisms between the interaction of H_2_O_2_ and ROS are needed much work to uncover in fibre development.

Auxin is necessary for fibre cell differentiation (Seagull and Giavalis, 2004). At the fibre initiation stage, specific and ectopic expression of the IAA biosynthetic gene *iaaM* driven by the promoter of *Floral Binding Protein7* (*FBP7*) significantly increased IAA accumulation in the epidermis of ovules and fibre numbers (Zhang *et al*., 2011). Excess transcripts of *GhPIN3* in the outer integument promoted fibre‐specific auxin accumulation for fibre initiation, indicating that all the auxin signalling/pathway components play important roles and have a division of labour in fibre development (Zhang *et al*., 2017a). *In vitro* assays have shown the positive function of BR in fibre development (Sun *et al*., 2005). *GhDET2*, encoding the rate‐limiting enzyme steroid 5α‐reductase in BR synthesis, was shown a positive function in fibre density and length (Luo *et al*., 2007). The BR receptor BRI1 (brassinosteroid insensitive1) was cloned and the transgenic plants showed that GhBRI1 regulated cellulose deposition in the SCW and fibre maturation stages (Sun *et al*., 2004, 2015b). *PAGODA1* (*PAG1*) encodes CYP734A1 that degrades BRs via C‐26 hydroxylation to negatively regulate fibre development (Yang *et al*., 2014). As a type of classic acid protein, Gh14‐3‐3 can interact with GhBZR1 to modulate BR signalling and promote fibre initiation and elongation (Zhou *et al*., 2015). All these evidences associated with BR metabolism and signalling factors in fibre development displayed a consistent and clear regulation pathway involving BR (Figure 4).

MADS‐box containing genes are a superfamily in plants, and several MADS transcription factors have been identified in cotton. GhMADS11 can promote yeast cell elongation; while over‐expression of GhMADS14 decreased the GA content and hypocotyl length in Arabidopsis, suggesting a possible GA pathway involving GhMADS14 in fibre development (Li *et al*., 2011; Zhou *et al*., 2014). From *G. barbadense*, a TCP encoding gene was identified with higher expression in fibre elongation; a gene ChIP assay and physiological analysis showed that GbTCP positively regulated GA synthesis to mediate fibre development (Hao *et al*., 2012). Moreover, GhTCP14 was also identified to have higher transcripts in fibre initiation and elongation from *G. hirsutum*, ectopic expression of which enhanced trichomes on stem, inflorescence, and root as well as the auxin distribution in Arabidopsis. An electrophoretic mobility shift assay (EMSA) showed the binding between GhTCP14 and the promoters of AUX1, IAA3 and PIN2, some key genes in the auxin pathway (Wang *et al*., 2013). All above results showed that TCP was involved in several phytohormone pathways to regulate fibre development (Figure 4).

Recently, many studies have shown that short peptides are a new type of plant hormone involved in different signalling pathways in plant growth and development, such as the well‐known CLAVATA3 (Ito *et al*., 2006; Kondo *et al*., 2006; Matsubayashi, 2018; Matsubayashi and Sakagami, 2006; Nakaminami *et al*., 2018). As a type of novel peptide hormone, phytosulfokine‐α (PSK‐α) has been shown to promote cell proliferation and differentiation during different plant development stages including callus growth, root growth and hypocotyl elongation (Igasaki *et al*., 2003; Kutschmar *et al*., 2009; Matsubayashi and Sakagami, 2006; Stuhrwohldt *et al*., 2011; Yang *et al*., 2001). In cotton, *in vitro* culture of cotton ovules with external PSK‐α promoted fibre cell elongation, and the longest fibres were observed under combined application of GA_3_ + IAA + PSK‐α. Thus, it is credible that PSK‐α and IAA together display synergistic and positive effect in fibre elongation.

Moreover, fibre cells fail to develop without GA_3_, which illustrates that the promotion of cotton fibre elongation by PSK‐α is dependent on GA_3_. Through a transgenic approach, *GhPSK*, encoding a PSK precursor peptide, is over‐expressed and improves cotton fibre length and micronaire (Han *et al*., 2014). These findings indicate that PSK acts as a novel peptide signal in cotton fibre development regulation, which interacts with auxin and GA_3_ during fibre cell development, and may also regulate crosstalk between auxin and GA. Nevertheless, it is unknown whether interaction among PSK‐α and other phytohormones (e.g. ethylene, CK) occurs and the underlying molecular mechanisms are unclear. Different techniques or approaches should be employed to identify the potential chemical or signal molecules involved in fibre cell development.

## The mechanisms of epigenetic modifications in cotton fibre development

Epigenetic modifications are an important regulator in plant development, but research on the epigenetics of cotton fibre development has been scarce until now. Histone deacetylase (HDA) decreases histone and nonhistone protein acetylation levels at lysines and is involved in the gene transcription or protein function. GhHDA5 is preferentially expressed at the fibre initiation stage (−1 and 0 DPA). GhHDA5 RNAi lines changed the ROS homeostasis and elevated autophagic cell death, which, in turn, decreased fibre initiation and lint yield. ChIP‐PCR showed that H3K9 acetylation level on some associated downstream genes regulated by GhHDA5 was up‐regulated in the RNAi lines (Kumar *et al*., 2018). HISTONE MONOUBIQUITINATION2 (HUB2) encoding histone H2B monoubiquitination E3 ligase was shown to have a role in fibre elongation and SCW deposition in transgenic cotton. Moreover, a key transcriptional repressor, GhKNL1, was ubiquitinated and degraded directly via the ubiquitin‐26S proteasome associated with GhHUB2, which promoted downstream gene expression, fibre elongation and SCW deposition (Feng *et al*., 2018; Figure 4).

As a kind of noncoding RNA, microRNAs such as miR828 and miR858 have been confirmed to regulate fibre development *via* targeting MYB2 homeologs in *G. hirsutum* (Guan *et al*., 2014). Another study also unravelled the key roles of miRNA156/157 for fibre elongation in *G. barbadense* (Liu *et al*., 2014b). Furthermore, the bidirectional transcript of GhMML3_A12 generated the siRNA involved in cotton fibre initiation (Wan *et al*., 2016). Using the fibreless mutant xuzhou142*fl*, RNA was extracted from epidermal cells of ovules to perform high‐throughput RNA‐seq. The lncRNAs and circular RNAs were identified, and of these, 645 and 651 lncRNAs were preferentially expressed in the fibreless and fibre‐attached lines, respectively. *Via* a virus‐induced gene silencing (VIGS) approach, down‐regulation of three lncRNAs increased the fibre initial numbers significantly (Hu *et al*., 2018). Another study also identified more than 35 000 lncRNAs and showed their potential functions in fibre development of *G. barbadense* (Wang *et al*., 2015a,2015b). Aforementioned evidences show that various noncoding RNAs may be involved in fibre development through some novel pathways and provide us more original knowledge about fibre development.

Through a genome‐wide DNA methylation assay, increased DNA methylation was found in fibre development, which was regulated by an active H3K9me2‐dependent pathway rather than the RNA‐directed DNA methylation (RdDM) pathway. Further multi‐omics analysis uncovered that in fibre differentiation, DNA methylation played a role through regulating lipid biosynthesis and ROS metabolism (Wang *et al*., 2016a; Figure 3). A variety of epigenetic modifications are involved in fibre development, and some downstream pathways have been studied and revealed. The possible interactive relationships among these epigenetic factors are still unknown. Application of some advanced molecular technologies such as high‐throughput chromosome conformation capture (Hi‐C) would be helpful for further understanding of the complex epigenetic regulation web in plant trichome development.

## The primary and secondary metabolism involved in cotton fibre development

Metabolites and inorganic ions are necessary for plant life and perform diverse functions in plant development as signals, cofactors and structural components (Abraham *et al*., 2016; Bhandari *et al*., 2015; Fiehn *et al*., 2000; Kopka *et al*., 2004; Rennenberg and Herschbach, 2014). Through transcriptional analysis of xuzhou142*fl*, the classic glabrous cotton mutant, very‐long‐chain fatty acids (VLCFAs) were identified to regulate the ethylene pathway involved in fibre development (Qin *et al*., 2007; Shi *et al*., 2006). Thus, lipids play important roles in fibre development. Deng *et al*. (2016) also reported that GhLTPG1, a GPI‐anchored lipid transport protein, bound and transported phosphatidylinositol mono‐phosphates to promote fibre elongation.

Sucrose is a translocated sugar that affects energy conservation and is believed to have a vital role in cellulose biosynthesis in fibre development (Amor *et al*., 1995). Moreover, sucrose is a unique carbon source from photosynthetic assimilation for fibre development, which has two necessary roles in fibre elongation. First, sucrose can be catalysed into UDP‐glucose to function as the direct substrate for cellulose synthesis in fibre development (Ruan *et al*., 1997, 2003; Zhang *et al*., 2017b). Second, sucrose and its hexose derivatives are considered to be involved in turgor pressure regulation during fibre cell expansion (Ruan, 2007). Over‐expression of a spinach sucrose‐phosphate synthase (SPS) significantly increased secondary wall thickness and fibre quality (Haigler *et al*., 2007). Sucrose synthase (SUS) reversibly catalyses sucrose synthesis and cleavage. Over‐expression of a potato SUS in cotton advanced leaf expansion, repressed seed abortion and promoted fibre production (Xu *et al*., 2012), which all show that ectopic expression of SUS and SPS in cotton has positive effects on fibre development.

Using an antisense method, down‐regulation of internal SUS gene expression also reduced cotton fibre cell initiation, elongation and seed development accordingly (Ruan *et al*., 2003). Using population genetics, *GhsusA1* was identified as tightly correlated with fibre quality. Subsequently, genetic analysis mapped *GhsusA1* to homoeologous subgenomes A8 and D8. Moreover, transgenic cotton showed that over‐expression of *GhsusA1* increased fibre length, strength, and thickness along with up‐regulated transcripts and enzyme activity of *GhsusA1* (Jiang *et al*., 2012). These results indicated the important roles of sucrose metabolism in fibre development.

Both potassium (K^+^) and calcium (Ca^2+^) play critical roles in cotton fibre cell development through regulating the cell turgor and shape as well as cell wall structure morphogenesis (Qin and Zhu, 2011; Tang *et al*., 2014a,2014b; Wang and Ruan, 2013; Yang *et al*., 2016a,2016b). Over‐expressing *AnnGh3*, which encodes an annexin with Ca^2+^‐binding ability, increased trichome initiation and length on leaves of transgenic Arabidopsis, suggesting the potential positive role of *AnnGh3* in fibre cell initiation and elongation of cotton (Li *et al*., 2013a,2013b). Another annexin, *GhAnn2,* was also shown to regulate fibre elongation and SCW through mediating Ca^2+^ dynamics and signalling in cotton (Tang *et al*., 2014b). GhCaM7, a calcium sensor, might regulate ROS accumulation and act as a molecular linker between Ca^2+^ and ROS signal pathways in early fibre development (Tang *et al*., 2014a).

Previous studies showed that ROS regulated fibre development in a manner dependent on Ca^2+^ content (Shao *et al*., 2011; Tang *et al*., 2014a). Another study also showed that an optimum increase in ROS‐induced sucrose transporters (GhSUT1 and GhSUT2‐A) and K^+^ transporters (GhKT1 and GhKT2) to promote fibre elongation (Guo *et al*., 2016). A recent study shed some light on the interaction of K^+^ and Ca^2+^ in fibre cell elongation. Ca^2+^ deficiency combined with modest K^*+*^ (0 mm Ca^2+^ and 27 mm K^+^) played a positive role in fibre elongation *in vitro*. Further, the transgenic plants showed that *CBL‐INTERACTING PROTEIN KINASE6* (*GhCIPK6*) mediated the uptake of K^+^ in a Ca^2+^‐deficient manner, clarifying the relationship between Ca^2+^, K^+^ and fibre elongation (Guo *et al*., 2017; Figure 5). From the above findings, Ca^2+^ has been shown to play multiple roles through mediating different metabolic pathways in fibre development, which may be dependent on its specific concentration gradients in different subcellular compartments.

Expansin, a type of glycoprotein, is the first cell wall‐loosening protein identified that functions without any evident hydrolytic cleavage or other enzymatic activity (Mcqueen‐Mason *et al*., 1992). Expansin is also the only protein that can induce cell wall expansion *in vitro*. Cotton fibre is one of the longest plant cells, with cell lengths thousands of times their width (Kim and Triplett, 2001), indicating that fibre cells have distinct cell expansibility. GbEXPATR, a species‐specific α‐expansin, enhances cotton fibre elongation by manipulating cell wall composition and physical properties and may be a vital and fundamental regulator in the fibre cell elongation (Li *et al*., 2016). However, the direct upstream regulation factors or interaction factors of expansin are still ambiguous. Yeast two‐hybrid screening or protein immunoprecipitation approaches would be useful to identify the potential and direct interaction factors, to fill in some gaps between upstream and downstream factors in trichome development.

## Progress on trichome development in other species

As a general and multifunctional plant organ, trichomes have also been studied in many other plant species. In cucumber (*Cucumis sativus*), several mutants with abnormal trichomes such as *trichome‐less* (*tril*), *glabrous 3* (*csgl3*) (Wang *et al*., 2016c), *tiny branched hair* (*tbh*) (Pan *et al*., 2015), *micro‐trichome* (*mict*) (Chen *et al*., 2014) and *glabrous 1* (*csgl1*) (Zhao *et al*., 2015) have been identified and studied. Previous work indicates that CsGL3, TRIL, MICT, TBH and CsGL1 encode HD‐Zip proteins in different subfamilies. *MICT*,* TBH* and *CsGLI* are allelic with alternative splicing. *CsGL3* and *TRIL* are allelic and epistatic to *TBH*,* MICT* and *CsGL1* on the regulation of trichome development. A positive regulator network for trichome development in cucumber was proposed and found to be different from that in Arabidopsis (Liu *et al*., 2016).

In tomato, the *WOOLLY* (*Wo*) gene, homolog to the Arabidopsis GL2, and a B‐type cyclin gene, *SlCycB2*, control the initiation and development of trichomes together (Yang *et al*., 2011a,2011b). Through a hairless mutant, a SRA1 (Specifically Rac1‐Associated protein) subunit of the WAVE regulatory complex was shown to be a prerequisite for trichome development of tomato, which indicates that proper actin‐cytoskeleton dynamics are necessary for normal trichome morphogenesis (Kang *et al*., 2016). In *A. annua*, over‐expressing a β‐glucosidase gene increased trichome density in leaf and flowers, and resulted in obvious enhancement of artemisinin content (Singh *et al*., 2016), which indicated that carbohydrate metabolism plays a key role in trichome cell differentiation in *A. annua*.

Over‐expressing a noncoding RNA‐miR156 precursor in alfalfa (*Medicago sativa* L.) down‐regulated three target genes (*SPL6*,* SPL12* and *SPL13*) transcripts and increased trichome numbers, which indicate the importance of epigenetic modification in trichome development of alfalfa (Aung *et al*., 2015).

Despite trichome research being deficient in other species, these studies provide supporting evidence for results from Arabidopsis and cotton and give original and valuable information about trichome development. Increasing evidence shows that trichome developmental regulation consists of complicated and diverse physiological pathways, in which some key genes play vital roles and can be engineered to modify the trichome morphological structure artificially.

## Comparison of the molecular mechanisms between Arabidopsis and cotton in trichome development

The molecular mechanisms in trichome development of Arabidopsis and cotton are well understood and indicate that Arabidopsis and cotton evolved different trichome development patterns. In Arabidopsis, the trichomes are a type of single‐cell organ with several branches. In cotton, the trichomes are a type of single‐cell organ without any branches. However, trichome length is longer on the cotton seed coat than in Arabidopsis leaf. These morphological dissimilarities indicate different underlying mechanisms. The reason for the morphological dissimilarities may be that branches in Arabidopsis trichomes contribute to the more contact area between plant and environment, so as to enhance the role of protection belt; however, cotton fibre in the boll does not contact the external environment directly and just acts as factory for cellulose, which profit from the no‐branched and longer cell type. Asymmetric subgenome gene expression and *cis*‐regulatory differentiation during cotton domestication also indicate the more complicated regulation network in fibre development (Wang *et al*., 2017a). Many MYBMIXTA and HD‐ZIP IV type transcription factors have shown conserved and vital roles in the regulation of different trichome development stages. In Arabidopsis, the necessary hub consisting of GL1, GL3, TTG1 and EGL1 is a general controller, which functions directly with GL2 to initiate trichome formation. The homologs of hub transcription factors (GL1, GL3, TTG1 and EGL1) in cotton were not identified as a central hub, although they played some important roles in fibre initiation or elongation (Tables 1 and 2).

**Table 1:** Key transcription factors in the regulation of Arabidopsis trichome development

Gene name	Gene locus	Gene family	Function in trichome	References
GL1	AT3G27920	MYB	Positive and negative in initiation	Larkin *et al*. (1994)
GL2	AT1G79840	HD‐ZIP IV	Positive in initiation	Johnson *et al*. (2002)
GL3	AT5G41315	bHLH	Positive in initiation and branching	Payne *et al*. (2000); Shen *et al*. (2006)
EGL3	AT1G63650	bHLH	Positive in initiation	Zhang *et al*. (2003)
TTG1	AT5G24520	WD repeat	Positive in initiation	Payne *et al*. (2000)
TTG2	AT2G37260	WRKY	Positive in initiation	Johnson *et al*. (2002)
TRY	AT5G53200	MYB	Negative in initiation	Schellmann *et al*. (2002); Kirik *et al*. (2004)
CPC	AT2G46410	MYB	Negative in initiation	Schellmann *et al*. (2002); Kirik *et al*. (2004)
GIS	AT3G58070	C2H2	Positive in initiation and negative in branching	An *et al*. (2012)
GIS2	AT5G06650	C2H2	Positive in initiation	Gan *et al*. (2007)
GIS3	AT1G68360	C2H2	Positive in initiation	Sun *et al*. (2015a)
ZFP8	AT2G41940	C2H2/zinc finger	Positive in initiation	Gan *et al*. (2007)
TEM1	AT1G25560	AP2/B3	Negative in initiation	Matias‐Hernandez *et al*. (2016)
TEM2	AT1G68840	AP2/B3	Negative in initiation	Matias‐Hernandez *et al*. (2016)
TCP4	AT3G15030	TCP	Negative in trichome branching	Vadde *et al*. (2018)
JAZ1	AT1G19180	JASMONATE‐ZIM‐DOMAIN	Negative in initiation	Qi *et al*. (2011)
GAI	AT1G14920	DELLAR	Negative in initiation	Jacobsen *et al*. (1996)

**Table 2:** The key transcription factors in fibre development of cotton (*Gossypium hirsutum*)

Gene name	Gene locus	Gen family	Function in fibre	References
MYB109	Gh_A05G3123	MYB	Positive in initiation and elongation	Suo *et al*. (2003)
MYB25	Gh_D04G1901	MYB	Positive in leaf trichome and fibre initiation	Machado *et al*. (2009)
MML3_A12	Gh_A12G1503	MYB	Positive in fuzz fibre initiation	Wan *et al*. (2016)
MML4_D12	Gh_D12G1630	MYB	Positive in lint fibre initiation	Wu *et al*. (2018)
MYB212	Gh_D11G3078	MYB	Positive in elongation	Sun *et al*. (2018)
MYB46_D13	Gh_D13G2261	MYB	Positive in secondary cell wall deposition	Huang *et al*. (2017)
MYB46_D9	Gh_D09G1082	MYB	Positive in secondary cell wall deposition	Huang *et al*. (2017)
CPC	Gh_D06G2201	MYB	Negative in initiation	Liu *et al*. (2015a)
TRY	Gh_A11G0869	MYB	Negative in initiation	Liu *et al*. (2015a)
HD1	Gh_D06G1607	HD‐ZIP IV	Positive in initiation	Zhang *et al*. (2010)
HOX3	Gh_A05G3845	HD‐ZIP IV	Positive in initiation	Zhang *et al*. (2010)
TTG1	Gh_A08G0926	WD repeat	Positive in initiation	Liu *et al*. (2015b)
TTG2	Gh_A10G1120	WD repeat	Positive in initiation	Liu *et al*. (2015b)
TTG3	Gh_Sca011289G01	WD repeat	Positive in initiation	Liu *et al*. (2015b)
TTG4	Gh_D02G1136	WD repeat	Positive in initiation	Liu *et al*. (2015b)
SLR1	Gh_A07G0717	DELLA	Negative in elongation	Shan *et al*. (2014)
BZR1	Gh_A05G1683	BES1_N	Positive in initiation	Zhou *et al*. (2015)
MADS11	Gh_A03G0634	MADS‐box	Positive in elongation	Li *et al*. (2011)
MADS14	Gh_A05G2136	MADS‐box	Negative in fibre elongation	Zhou *et al*. (2014)
TCP14	Gh_A11G0279	TCP	Positive in initiation and elongation	Wang *et al*. (2013)
JAZ2	Gh_D06G0810	JASMONATE‐ZIN‐DOMAIN	Negative in lint and fuzz fibre initiation	Hu *et al*. (2016)
FSN1	Gh_A12G1049	NAC	Positive in SCW	Zhang *et al*. (2018a,2018b)

In addition, many more factors associated with cell wall morphology (e.g. GbEXPATR, GhEXP1) (Li *et al*., 2016; Orford and Timmis, 1998) were shown to have important roles in fibre development, which is tightly related to the greater length and higher content of cellulose in cotton fibre. The phytohormones ABA and auxin play important roles in plant development. Whether ABA functions in trichome development of Arabidopsis is unknown, but it plays negative roles in fibre initiation of cotton (Chen *et al*., 1997; Dhindsa *et al*., 1976; Li *et al*., 2015b). Similarly, auxin has been identified as a key promoter of fibre development, but whether it also plays roles in Arabidopsis trichome development has been unclear until now (Table 3). The common MYB, bHLH and WD repeat type transcription factors may be conserved and older elements, while other factors associated with particular phytohormones (e.g. auxin, ABA), epigenetic modifications or specific metabolisms may be younger and more specific in the evolution of plant trichome development.

**Table 3:** Comparison of the molecular mechanisms between Arabidopsis trichome and cotton fibre development

	Species	Arabidopsis		Cotton	
		Development stages			
Classification	Types	Trichome initiation	Trichome branching	Fibre initiation and elongation	Secondary cell wall synthesis
Transcription factors	MYB	GL1, TRY, CPC	NR	MYB25, MYB109, MYB212, CPC, TRY	MYB46
	HD‐ZIP	GL2	NR	HD1, HOX3	NR
	bHLH	GL3, EGL3	GL3	NR	NR
	WD repeat	TTG1	NR	TTG1, TTG2, TTG3, TTG4	NR
	Other	TEM1/2, GISs, TTG2	GIS, TCP4	TCP14, MADS11/14	FSN1
Phytohormones	Auxin	NR	NR	Positive (AUX1, PIN3)	NR
	GA	Positive	NR	Positive (SLR1)	NR
	JA	Positive (JAZ1)	NR	Positive (JAZ2)	NR
	Ethylene	NR	NR	Positive (ACO1)	NR
	BR	NR	NR	Positive (DET2, PAG1, BZR1)	NR
	ABA	NR	NR	Negative	NR
Epigenetic modifications	DNA methylation	NR	NR	Negative	NR
	RNA modification	Positive (ECT2)	NR	NR	NR
	Histone methylation	NR	NR	NR	NR
	Histone acetylation	Positive (GCN5)	Negative (GCN5)	Positive (HDA5)	NR
	Protein ubiquitination	NR	Negative (UPL3)	Positive (HUB2)	Positive (HUB2)
	Noncoding RNA	NR	Negative (miR319)	Positive (GhMML3_A12, miRNA156/157, miR828/858)	NR

NR, not reported.

**Figure 2:**
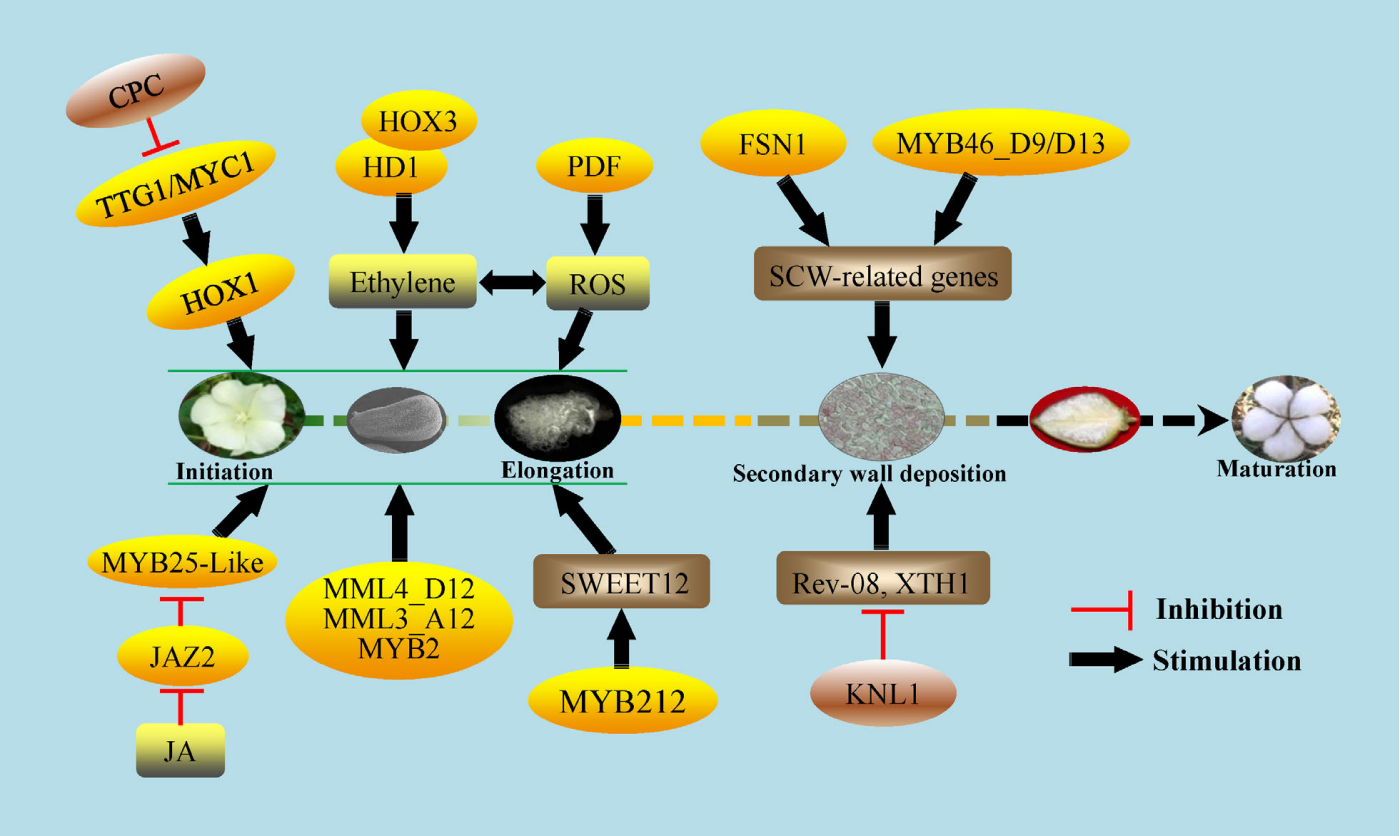
Key transcription factors in the regulation of cotton fibre development. The conserved transcriptional factors associated with MYB, bHLH and HD‐bZIP types were identified, and the possible mechanisms are presented in the regulation network of fibre development. The key positive and negative transcription factors are represented in yellow and brown ovals. The grey rounded rectangles indicate the phytohormones JA, ethylene and ROS. Most of them function in fibre (lint and fuzz) initiation and elongation except FSN, MYB46_D9/D13 and KNL1, which function in the secondary cell wall deposition stage. CPC functions upstream of TTG1/MYC1 as a negative factor similar with that in Arabidopsis. KNL1 is a transcription repressor to inhibit the expression of cell wall and SCW‐related genes. During the maturation stage, the underlying molecular mechanisms and factors are mostly unknown, in which stage the fibres start dehydrating and become dry. Clarification for fibre maturation may contribute to understanding the premature mechanisms in cotton.

**Figure 3:**
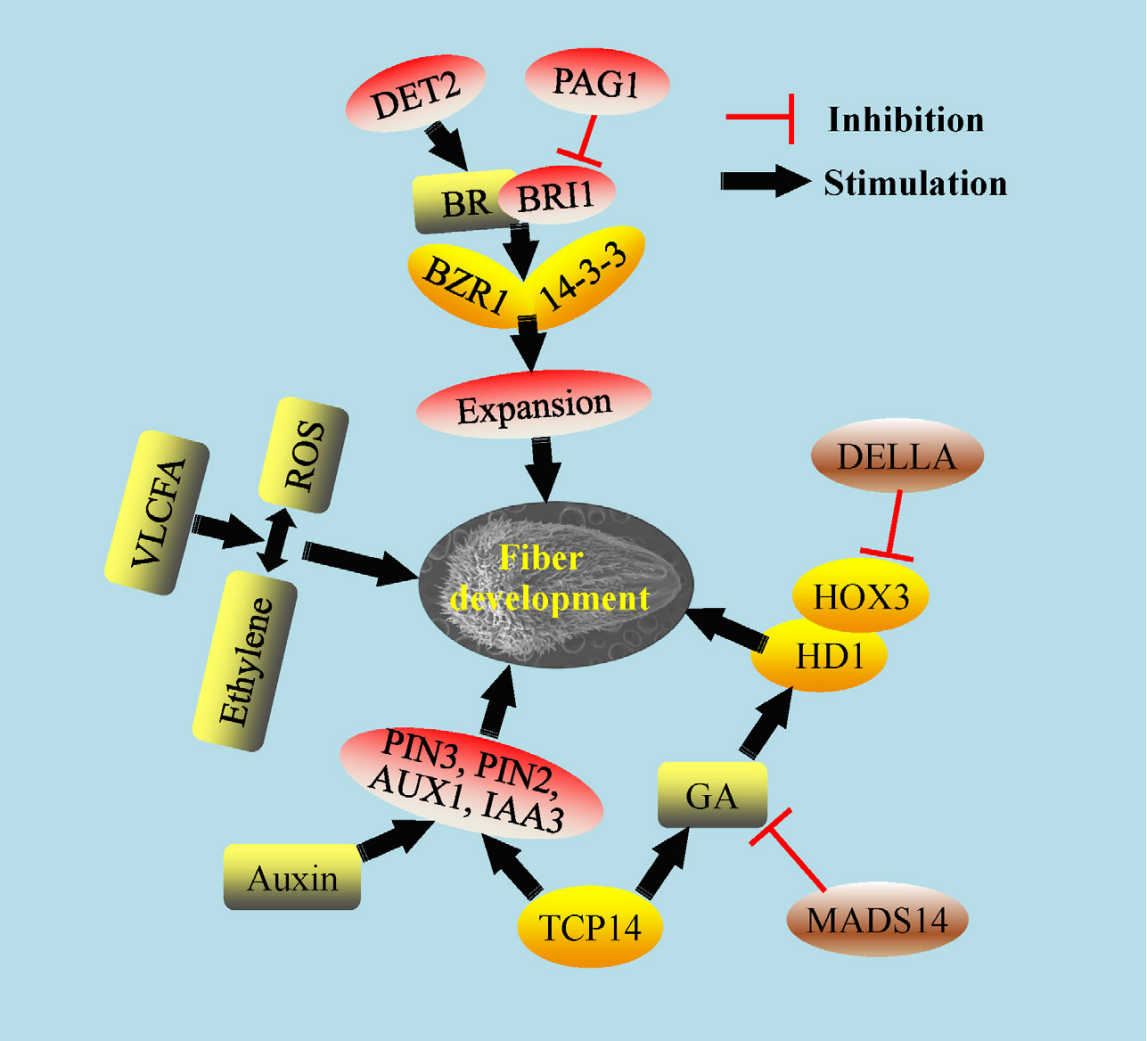
The molecular mechanisms of phytohormones in regulation of fibre development. The different phytohormones are involved in distinct pathways and molecular mechanisms in fibre development. Furthermore, some interactions among specific phytohormones and transcription factors are displayed. Yellow and brown ovals indicate the associated positive and negative transcription factors involved in the phytohormones, respectively. Grey rounded rectangles indicate the important phytohormones and metabolites. Other important proteins, receptor and enzymes are indicated with red and white ovals (e.g. PIN3, DET2, PAG1, BRI1).

**Figure 4:**
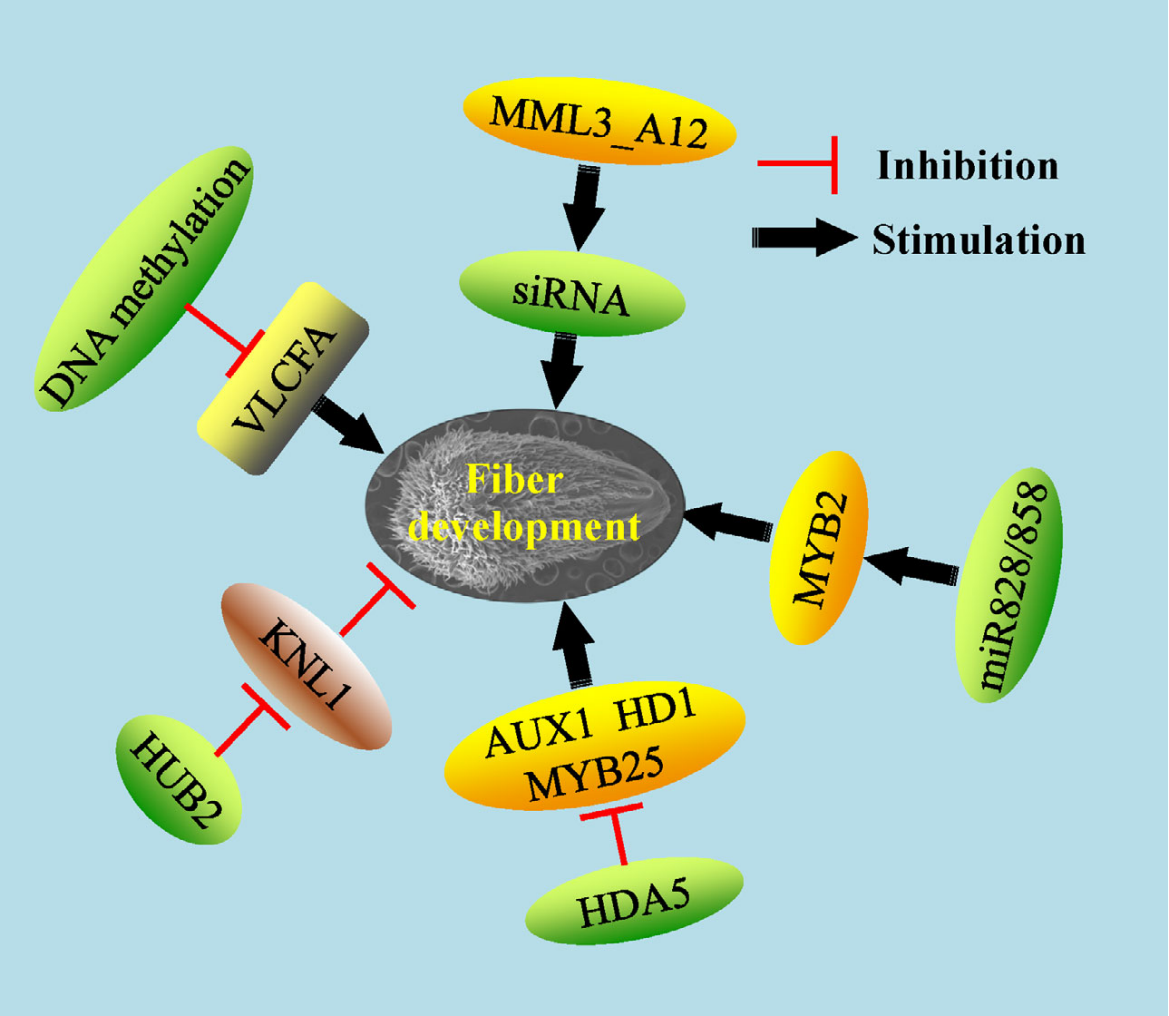
The epigenetic mechanisms in regulation of fibre development. Some epigenetic modifications involve distinct pathways and molecular mechanisms in fibre development. Additionally, some interactions among specific epigenetic modifications and other factors are presented here. Green ovals indicate the epigenetic modification factors. Yellow and brown ovals indicate the positive and negative transcription factors involved in the epigenetic modifications, respectively. Grey rounded rectangle indicates the associated metabolites.

**Figure 5:**
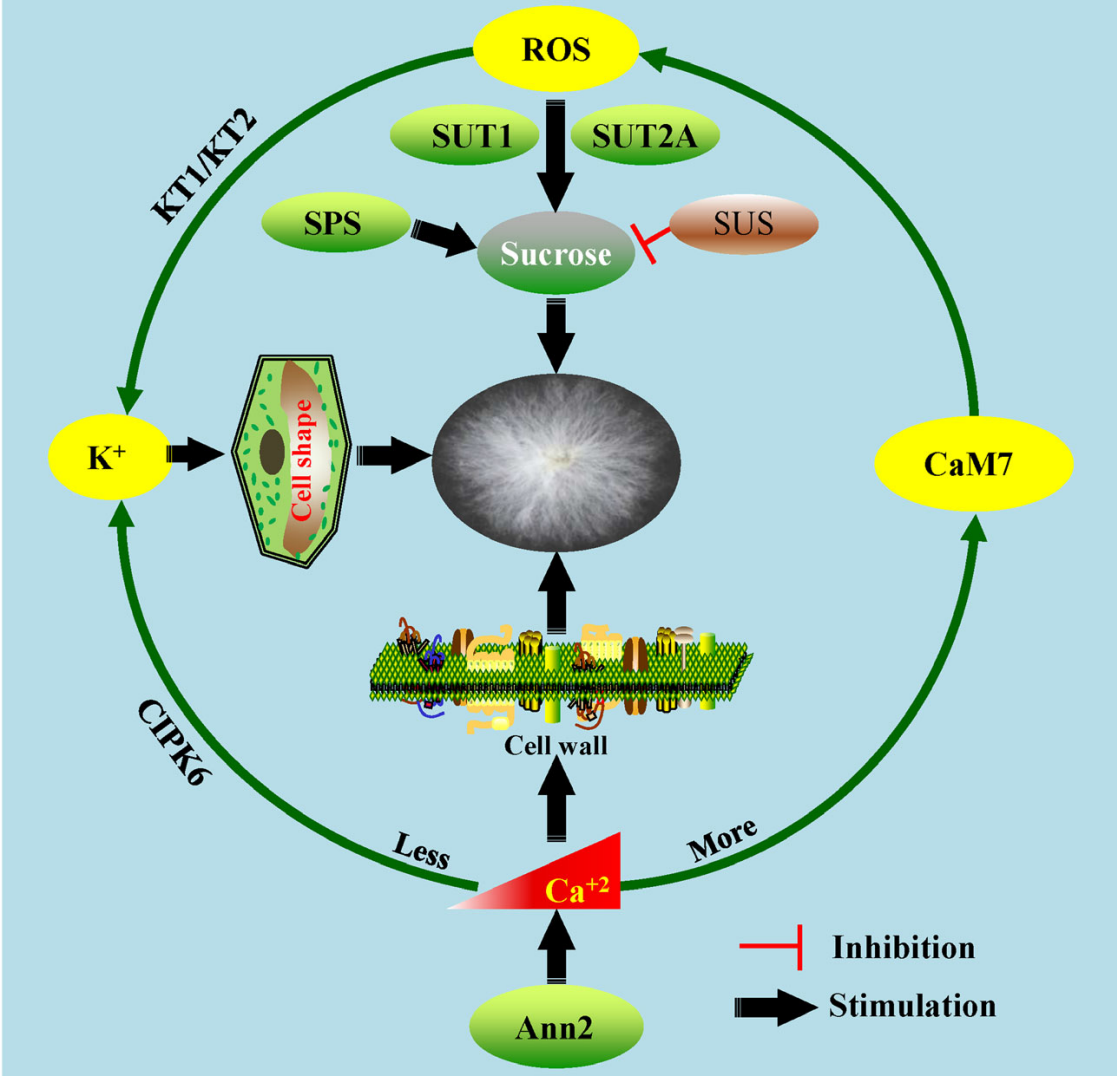
The interaction between metabolic pathways and signalling involving reactive oxygen species, K^+^ and Ca^+2^ in fibre development. ROS, K^+^ and Ca^+2^ play positive roles and mutually regulate fibre development. Ca^2+^ deficiency (whitening) induces K^+^ accumulation through CIPK6 to regulate fibre development; on the other hand, excessive Ca^2+^ (reddening) promotes calcium sensor CaM7 function and ROS accumulation, and increasing ROS also facilitate the K^+^ accumulation by KT1/KT2 function. Furthermore, sucrose functions downstream of ROS to involve the fibre development. Therefore, the homeostasis of K^+^ and Ca^+2^ contents in the cell is vital for fibre development. SPS (sucrose‐phosphate synthase) and SUS (sucrose synthase) regulate the sucrose synthesis and degradation, respectively. An annexin, GhAnn2, function upstream of the Ca^2+^ pathway in mediating fibre development.

It is becoming common knowledge that transcription factors can function upstream of metabolic pathways as structural regulators (Iwase *et al*., 2009). The regulation network of fibres (Figures 2, 3, 4, 5) shows that most MYB family transcription factors are upstream of the secondary metabolism pathways and phytohormones (e.g. ROS, pectin, ethylene) as in previous reports (Iwase *et al*., 2009), and downstream of epigenetic modifications (e.g. histone modification, microRNA regulation), except GhMML3_A12. Other transcriptional factors such as HD‐ZIP IV type (e.g. HD1, HOX3) are downstream of phytohormones, indicating complex regulation in cotton fibre development. Some glabrous mutants of cotton such as xuzhou142*fl* and MD17 all showed fibreless or fuzzless seeds but normal trichomes on leaf and stem, which further supported the different regulation model between the trichomes on vegetative (e.g. leaf, stem) and reproductive organs (e.g. ovule, seed) in plants (Liu *et al*., 2015b).

In plant cells, filamentous actin arrays and associated actin‐binding proteins are essential for proper cell morphogenesis as well as all kinds of cellular processes (Hussey *et al*., 2006; Staiger and Blanchoin, 2006). It was reported that GhACT1, an actin encoding gene, was vital for fibre elongation, and a kinesin isoform (GhKCH1) was involved in fibre cell growth through organizing the actin network and microtubule array (Li *et al*., 2005b; Preuss *et al*., 2004). Actin depolymerizing factor (ADF) is one of actin‐binding proteins involved in the regulation of actin‐cytoskeleton dynamics. GhADF1 was shown to play important roles in regulating fibre elongation as well as cellulose deposition of SCW in cotton fibres (Wang *et al*., 2009). However, the interactions among actin, ADF and KCH have not been uncovered in fibre development. Some biochemical or genetic studies of these genes should be carried out to explore their possible internal relationship in the actin dynamics of fibre development.

Unlike the sole transparent trichome cells on Arabidopsis leaf or stem, cotton fibres are colourful including white, brown and green. In contrast with white cotton, naturally coloured cotton (NCC) economizes dyeing and bleaching steps during textile processing, which obviously decreases the release of harmful pollutants and water costs, as well as effectively enhances fabric yield and quality (Efe *et al*., 2009; Hua *et al*., 2009). NCC are formed by synthesizing and accumulating natural pigments during the course of fibre development in the field. However, the pigments’ synthesis and accumulation are always negatively correlated with fibre quality and yield in NCC fibres (Efe *et al*., 2009; Feng *et al*., 2015; Hua *et al*., 2009; Zhang *et al*., 2009b).

Some progress has been made for the underlying mechanisms of brown fibre development. As a kind of condensed tannin, proanthocyanidins (PAs) are an important resource for the brown pigmentation in fibre (Hinchliffe *et al*., 2016; Nam *et al*., 2016). Previous studies have elucidated an MYB‐type transcription factor, TRANSPARENT TESTA 2 (TT2), that regulates PA biosynthesis and seed coat colouring specifically in Arabidopsis. Further, many TT2 homologs have also been shown to boost PA biosynthesis in species including poplar (*Populus tremula × P. tremuloides*), apple (*Malus × domestica*), cacao (*Theobroma cacao*), strawberry (*Fragaria × ananassa*) and *Lotus japonicus* (Gesell *et al*., 2014; Liu *et al*., 2014a, 2015b; Mellway *et al*., 2009; Schaart *et al*., 2013; Wang *et al*., 2017b; Yoshida *et al*., 2008), suggesting the conserved and key role of TT2 in PA regulation in higher plants.

In cotton, six loci (Lc1–Lc6) mediating brown fibre have been identified, and fine‐mapping confirmed the Lc1 gene as GhTT2‐3A, which triggers brown fibre formation by activating transcription of downstream PA pathway genes and accumulation of PA (Hinchliffe *et al*., 2016; Wang *et al*., 2014a; Yan *et al*., 2018). So, similar function and metabolism pathways of PA occur in the cotton fibre and Arabidopsis seed coat but not leaf trichomes, which indicated a likely orchestrated interaction web among fibre and seed development in cotton. Next, scientists should try to identify other upstream regulators for fibre and seed colouring such as transcription factors, epigenetic modifiers and so on to reveal the specific colouring mechanisms in trichomes on the seed coat of cotton.

## Concluding remarks and future perspectives

Regulation of trichome development involves many transcriptional factors and metabolism pathways, despite the fact that they are an organ with very little volume in plants. Based on this review, there are three main conclusions about trichome development. First, many advances have been achieved in identifying positive factors and pathways in trichome development, whereas there is very limited knowledge about negative regulation mechanisms and key factors, except the well‐studied TEM1/2 transcription repressor in Arabidopsis (Matias‐Hernandez *et al*. 2016). Given that ABA may inhibit fibre development in cotton, the underlying factors and mechanisms are still unknown. Along with the quick advancement and effective application of gene editing techniques such as CRISPR‐CAS9 in plants including cotton (Li *et al*., 2018; Wang *et al*., 2018), disadvantageous and negative target genes would be more valuable and practical, because their direct knockdown *in planta* by gene editing would confer positive and favourable phenotypes in plants for humans. Therefore, much more research should be conducted to exploit the negative factors in trichome development. The important roles of different phytohormones in trichome development are well known, but the interaction among these phytohormones and underlying molecular mechanisms require further work to identify, which would also contribute to the identification and analysis of positive and negative factors in trichome development.

Second, although Arabidopsis is a classic model plant, many more advances in gene identification of associated molecular mechanisms have been made in cotton fibre than in Arabidopsis trichomes. In recent decades, minimal research progress has been made in Arabidopsis trichomes; conversely, the understanding of cotton fibre development has been made great progress, especially following release of the cotton genome sequence (Li *et al*., 2014, 2015a; Zhang *et al*., 2015). With the updated release of reference genome sequence of cotton (Wang *et al*., 2019a), revealing the fibre development mechanisms would be accelerated. This indicates that scientific research driven by realistic demand in crop production may be more powerful and effective and also indicates the potentially more complicated regulation web in cotton due to its larger allotetraploid genome. The advanced technologies of high‐throughput sequencing offer substantial contributions, but more effort is needed to uncover more key genes, such as trichome‐specific transcription factors, which can be engineered for favourable characters such as longer cotton fibre, more spearmint (*Mentha spicata*) terpene and higher effective biosynthesis of other secondary metabolites (Singh *et al*., 2016; Wang *et al*., 2004; Wang *et al*. 2016b). On the basis of the trichome‐specific genes, the corresponding promoters (e.g. *FBP7*,* E6*) are also very valuable for the engineering improvements in crop yield and quality (Colombo *et al*., 1997; John and Crow, 1992). So, identifying and applying the tissue‐specific promoters as well as trichome‐specific genes would be very profitable to plant development research and crop genetic advancement.

Third, previous hypotheses have suggested that to increase fibre yield and quality, boosting seed development could be an effective and achievable strategy (Ruan, 2013). Arabidopsis TTG2 influences not only trichome development but also tannin and mucilage production in the seed coat (Johnson *et al*., 2002); like TTG2, GL2 regulates not only trichome development but also seed oil content (Shen *et al*., 2006). Using antisense and over‐expression of *GhsusA1* in cotton, *GhsusA1* was shown to positively regulate the fibre quality as well as the biomass accumulation in vegetative growth, boll size and seed weight in reproductive development (Jiang *et al*., 2012). These results indicated that some key genes in basic metabolic pathways (e.g. carbohydrate metabolism) function in several different developmental stages such as leaf, trichome and seed development. Some research in tomato also supported a potential relationship between trichome and seed development. *Wo* encodes an HD‐Zip protein, over‐expression of which resulted in both trichome overproduction and embryo lethality in tomato (Yang *et al*., 2011a). These results indicated that some multifunctional factors may be involved in trichome and seed regulation simultaneously through different downstream targets, which implied a close and sophisticated interaction between trichome and seed development. These advancements also suggested that the different cofactors and dimensional regulation network associated with the multifunctional factors are a future focus, and pointed out some unprecedented research findings and aspects of plants such as cotton and willow, which produce trichomes on the seed coat.

In future studies, more plant species should be investigated, and research should be focused on the intrinsic connection and interaction between trichomes and other organs like seeds, and the identification of the key regulators associated with them, and discerning how they behave with changing environments or development. These foci will allow researchers to provide novel insights and gene resources for plant development and crop improvements. On the other hand, the innovation and application of cutting‐edge scientific technologies and experimental approaches (e.g. chloroplast genetic engineering, nanoparticle bombardment transformation, VIGS method by agroinoculation of cotton seeds) in biology would facilitate understanding of the molecular mechanisms in plant trichome development (Jin and Daniell, 2015; Jin *et al*., 2012; Zhang *et al*., 2018a,2018b; Zhao *et al*., 2017).

## Author contributions

Z.W. and F.L. conceived and drafted the manuscript; Z.Y. collaborated in the manuscript preparation. All authors revised and approved the final manuscript.
